# Salvage bortezomib–dexamethasone and high-dose melphalan (HDM) and autologous stem cell support (ASCT) in myeloma patients at first relapse after HDM with ASCT. A phase-2 trial

**DOI:** 10.1038/bmt.2015.125

**Published:** 2015-06-29

**Authors:** P Gimsing, Ø Hjertner, N Abildgaard, N F Andersen, T G Dahl, H Gregersen, T W Klausen, U-H Mellqvist, O Linder, R Lindås, N Tøffner Clausen, S Lenhoff

**Affiliations:** 1 Department of Hematology, Rigshospitalet, University of Copenhagen, Copenhagen, Denmark; 2 Department of Hematology, St. Olavs University Hospital, Norwegian University of Science and Technology (NTNU), Trondheim, Norway; 3 Department of Hematology, Odense University Hospital, University of Southern Denmark, Odense, Denmark; 4 Department of Hematology, Aarhus University Hospital, Aarhus, Denmark; 5 Department of Hematology, Rikshospitalet, Oslo, Norway; 6 Department of Hematology, Aalborg University Hospital, Denmark; 7 Department of Hematology, Herlev University Hospital, Herlev, Denmark; 8 Department of Hematology, Sahlgrenska University Hospital, Gothenborg, Sweden; 9 Department of Hematology, Örebro University Hospital, Örebro, Sweden; 10 Department of Hematology, Haukeland University Hospital, Bergen, Norway; 11 Department of Hematology, Skåne University Hospital, Lund, Sweden

## Abstract

Until recently, only retrospective studies had been published on salvage high-dose melphalan (HDM) with autologous stem cell ‘transplantation' (ASCT). In a prospective, nonrandomized phase-2 study, we treated 53 bortezomib-naïve patients with bortezomib–dexamethasone as induction and bortezomib included in the conditioning regimen along with the HDM. Median progression-free survival (PFS), time to next treatment (TNT) and overall survival (OS) after start of reinduction therapy were 21.6, 22.8 and 46.6 months, respectively. For 49 patients who completed salvage bortezomib–HDM(II) with ASCT, there was no significant difference of PFS and TNT after HDM (II) compared with after the initial HDM(I), and thus patients were their own controls (PFS (I: 20.1 vs II: 19.3 months (*P*=0.8)) or TNT (I: 24.4 vs II: 20.7 months (*P*=0.8)). No significant differences in the response rates after salvage ASCT compared with the initial ASCT. Bortezomib–HDM conditioning combo was feasible, and toxicity was as expected for patients treated with bortezomib and ASCT. In conclusion, in bortezomib-naïve patients treated at first relapse with salvage ASCT including bortezomib, PSF and TNT did not differ significantly from initial ASCT and median OS was almost 5.5 years with acceptable toxicity. A recent prospective randomized study confirms salvage ASCT to be an effective treatment.

## Introduction

With the introduction of high-dose melphalan (HDM) followed by autologous stem cell ‘transplantation' (ASCT) and the use of new agents, the outcomes of patients with multiple myeloma have steadily improved. However, multiple myeloma is still an incurable disease, and patients will eventually relapse after front-line therapy. Because of the fact that ASCT is a highly efficient therapy in a majority of patients, it is a widespread routine to harvest a sufficient number of stem cells for at least two transplantations. A second ASCT can then be offered to relapsing patients who are still considered young and fit enough for this treatment. However, only few reports of the results of a second ASCT in the relapse setting had been published and all studies were retrospective,^1, 2, 3, 4, 5, 6, 7^ as recently reviewed.^8^ Recently, the first prospective phase-3 study was published from the UK.^9^ The duration of disease control after salvage ASCT has usually been observed to be shorter than after the initial ASCT.

It is a general observation that the time from first ASCT to relapse has a great impact on the prognosis for both progression-free survival (PFS) after second-line treatment and for overall survival (OS).^10, 11, 12, 13, 14^ New drugs such as bortezomib and IMIDs, thalidomide and lenalidomide have improved response rate and response duration and are more efficient as second-line treatment than conventional chemotherapy.^15, 16^ Furthermore, there are indications that treatment with bortezomib may overcome the adverse prognostic importance of some of the high-risk cytogenetic aberrations in multiple myeloma like t(4;14).^17^

We conducted a prospective study to explore the efficacy of salvage high-dose therapy with ASCT at first symptomatic relapse preceded by induction therapy with bortezomib and dexamethasone and inclusion of bortezomib in the conditioning regimen of ASCT, which has also been addressed by the French group,^18^ the Italian group^19^ and most recently a Japanese group^20^ and was shown to be feasible in a phase-1/2 study.^21^

## Materials and methods

### Inclusion criteria

Patients with multiple myeloma at first symptomatic relapse, who had been treated with HDM followed by ASCT as first-line treatment, were eligible for inclusion, if they had preserved at least 2.0 × 10^6^ CD34^+^ frozen stem cells/kg body weight.

### Exclusion criteria

Patients were excluded if they had received former treatment with bortezomib, had neuropathy grade ⩾3 or had WHO performance status >3.

### Study design

The trial was a prospective nonrandomized phase-2 study.

### Study treatment

Three courses of intravenous bortezomib (Velcade) at a dose of 1.3 mg/m^2^ on days 1, 4, 8 and 11 and oral dexamethasone at a dose of 20 mg on days 1, 2, 4, 5, 8, 9, 11 and 12 were followed by HDM (200 mg/m^2^) on day −2 and intravenous bortezomib at a dose of 1.3 mg/m^2^ on days −5 and −2, and subsequent infusion of at least 2.0 × 10^6^ CD34^+^ stem cells on day 0. Prophylactic antiviral, antibacterial, and antifungal treatment and G-CSF were given according to local routine. Bortezomib dose was reduced to 1.0 mg/m^2^ and subsequently to 0.7 mg/m^2^ in the case of neurotoxicity according to the manufacturer's instructions. The primary end point was a comparison of the PFS after salvage HDM with stem cell support (ASCT) with PFS after first ASCT. Secondary end points were (1) to determine the tolerability of including bortezomib in the conditioning regimen with HDM; (2) to determine the response rates of the salvage ASCT according to IMWG criteria;^22^ and (3) to determine the time schedule for marrow regeneration (neutrophil and platelet recovery) after the second ASCT. Furthermore, the efficacy in patients with early relapse (within first year) after first ASCT vs later relapse was explored.

All patients signed a written informed consent before inclusion. The study was approved by the ethics committees and health authorities in all participating countries and conducted in accordance with the Helsinki declaration of 1975 and the Guidelines for Good Clinical Practice. This study was registered at www.clinicaltrials.gov as no. NCT00508209.

### Statistical analysis

OS, PFS (event: progression or death of any cause) and time to next treatment (TNT) after salvage ASCT all had censored observation and were analyzed using the Kaplan–Meier method and Cox proportional hazard models.

Unless otherwise mentioned, OS, TNT and PFS were calculated from the start of bortezomib treatment. To compare TNT and PFS after initial ASCT and salvage ASCT, we calculated the ratio between TNT after salvage and initial ASCT and PFS after salvage and initial ASCT. These ratios were analyzed using the Kaplan–Meier method.

The McNemar test using exact *P*-values was used for comparisons of paired ordinal variables.

All *P*-values were two-sided, and *P*-values below 0.05 were considered significant. R version 3.0.0 was used for all calculations (R foundation for statistical computing, Vienna, Austria).

## Results

**Figure 1:**
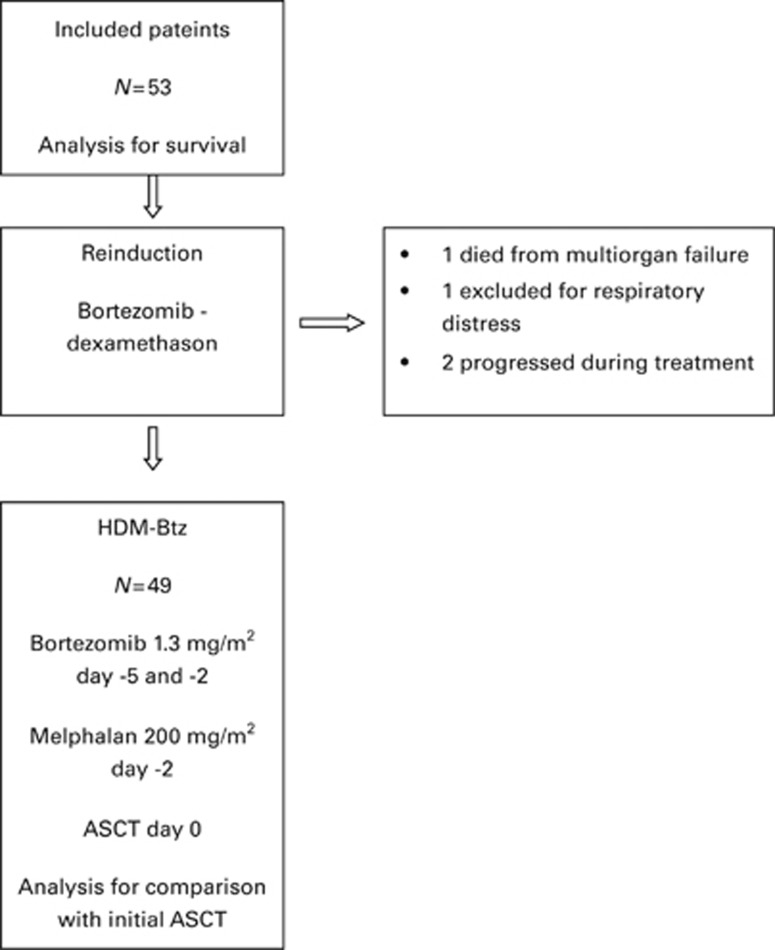
Phase 2 study of bortezomib–dexamethasone reinduction followed by bortezomib–high-dose melphalan (HDM-Btz) with autologous stem cell support (ASCT) at first relapse after initial ASCT. Survey of included patients.

Between 17 July 2007 and 8 June 2009, 53 patients with their first relapse after upfront HDM with ASCT were included, and the characteristics at inclusion are presented in Table 1. Consecutive patients fulfilling the inclusion criteria were included at each center. The initial induction therapy had been standard vincristine, doxorubicin, dexamethasone (VAD) or cyclophosphamide and dexamethasone (CTX/Dex), as previously described.^23^ No patient had received consolidation therapy, whereas seven patients had received Interferon-α 2b maintenance after the initial ASCT. Cytogenetics or fluorescence in situ hybridization (FISH) were not a part of the trial, and retospectively 36 patients had neither karyotype nor FISH results and therefore these results are not presented here. All patients received standard dose reinduction treatment with bortezomib and dexamethasone, but four patients never came to salvage HDM: one patient died from multiorgan failure after only one bortezomib injection, one patient developed respiratory distress syndrome and two patients developed progressive disease (see Figure 1). The median number of CD34^+^ stem cells given was 3.63 (range: 2.0–12.1) × 10^6^/kg body weight.

### Marrow regeneration

Time to neutrophils above 0.5–1.0 × 10^9^/L were 11 days (range 10–14) and 12 days (range 7–41), respectively. Time to platelets above 20–100 × 10^9^/L were 11 days (range 0–20) and 21 days (range 11–48), respectively. The overall median follow-up time was 30.4 months.

### Survival and response rates

**Figure 2:**
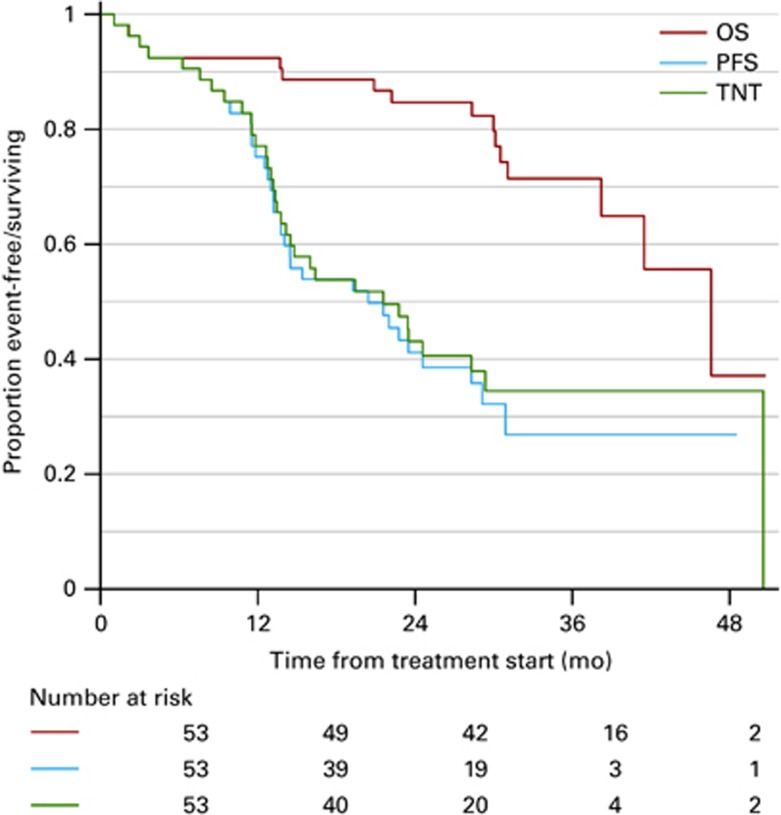
Kaplan–Meier plot of overall survival (OS), progression-free survival (PFS) and time to next treatment (TNT) from start of reinduction therapy of all included patients. The *Y* axis indicates the event-free survival (event=death or PD (PFS) or next treatment (TNT)).

The median follow-up times were 19.3, 19.4 and 30.9 months for median PFS, TNT and OS, respectively. The PFS, TNT and OS after the start of reinduction therapy were 21.6, 22.8 and 46.6 months, respectively (Figure 2). For the 49 patients who completed the salvage bortezomib–HDM with ASCT, the EFS, TNT and OS after ASCT were 19.3, 20.7 and 44.3 months, respectively. An updated survival from February 2015 with an overall follow-up time for OS of 51.1 months showed a median OS of 65.7 months (95% CI: 44.6;79.9).

The overall response rates were complete response/near complete response (CR/nCR) 32.1%, very good partial response (VGPR) 28.3%, partial response (PR) 26.4%, minor response (MR) 1.9%, progressive disease (PD) 3.8% and non-evaluable (NE) 3.8% for the 53 included patients. The response rates for the 49 patients who completed ASCT appear from Table 2 showing response rates before and after ASCT. Altogether, 35% of the patients achieved CR/nCR after salvage ASCT, whereas 22% of the patients achieved CR/nCR after initial ASCT. Nine patients (18%) had better response after salvage ASCT, and three patients (6%) had the best response after primary ASCT, although this difference was not significant (*P*=0.14, McNemar test).

### Response after initial induction therapy compared with response after reinduction

Eighteen patients improved the depth of response from the initial induction to the velcade–dex induction (9 to CR/nCR (4 from PR, 4 from SD and 1 from NE), 6 to VGPR (5 from PR and 1 from SD), 2 from SD to PR and 1 from PD to SD), whereas 10 patients had less deep responses (one from CR/nCR to PR, 8 from PR (6 to SD and 2 to PD) and 1 from SD to PD). Eleven patients had the same response as that after the initial induction, whereas response evaluation was not possible in four patients. There was a significant relationship between the CR/nCR and PFS (*P*=0.047), but not between overall response rate (PR or better) and PFS (*P*>0.13).

**Figure 3:**
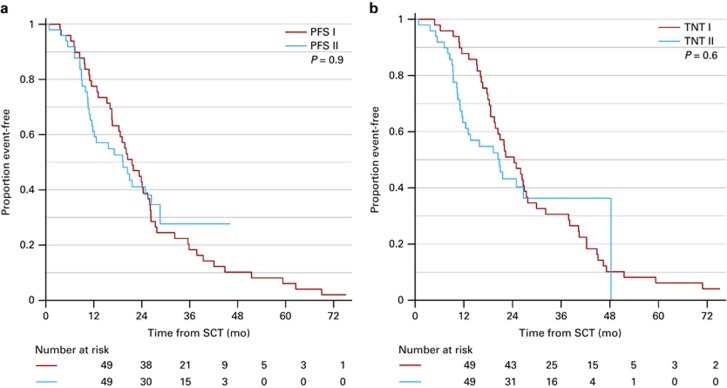
Kaplan–Meier Plot of PFS II after salvage autologous stem cell transplantation (ASCT) compared with PFS I after initial ASCT (**a**) and TNT II after salvage ASCT compared with TNT I after initial ASCT (**b**). The *Y* axis indicates the event-free survival (event=death or PD (PFS) (**a**) or next treatment (TNT) (**b**)).

Comparison between the salvage ASCT and the initial ASCT shows no significant difference of PFS (I: 20.1 vs II: 19.3 months (*P*=0.8)) or TNT (I: 24.4 vs II: 20.7 months (*P*=0.8)) (Figure 3). There was a significant correlation between PFS after the initial ASCT and the salvage ASCT (*P*=0.0005, Cox regression). Eleven patients, who had relapsed within the first year after initial ASCT, had median 10.0 months PFS compared with 24.4 months in patients with later relapse (*P*=0.009). Twenty-one patients, who had relapsed within the first 2 years after initial ASCT, had median 11.5 months PFS compared with 28.6 months in patients with later relapse (*P*=0.002). The median ratio between TNT after salvage and initial ASCT was 0.71 (CI: 0.60–0.92), and the median ratio between PFS after salvage and initial ASCT was 0.8 (CI: 0.64–1.108).

### Toxicity

Neurotoxicity data are presented in Table 3. About half of the patients had some degree of neurotoxicity after the induction therapy. In three patients, the neuropathy progressed after the combination of bortezomib and HDM. The neurological symptoms resolved in most of the patients after high-dose therapy. However, four patients had unresolved neuropathy (one patient with grade 3 neuropathic pain, one patient with grade 3 sensory neuropathy and two patients with combined neuropathic pain and sensory neuropathy). Although the symptoms disappeared in three of these patients within 6–12 months, one patient still had unchanged symptoms >1 year after ASCT. Non-neurological toxicity is presented in Table 4 and it did not differ from what is seen in other patients treated with HDM and ASCT.

## Discussion

In this prospective study, we demonstrate that reinduction with bortezomib and dexamethasone and addition of bortezomib to conditioning HDM is a feasible treatment with longer PFS than expected. By intention-to-treat analysis, the study population had a median PFS of about 20 months and an overall survival of almost 5.5 years after a second ASCT.

Richardson *et al.*^24^ showed in the APEX study that bortezomib single-drug treatment gave a PFS of 8.1 months in a bortezomib-naive population receiving second-line treatment, including two-thirds initially treated with stem cell transplantation or other high-dose regimens. Hjorth *et al.*^25^ found similar PFS for patients treated with bortezomib–dexamethasone (7.2 months) and thalidomide-dexamethasone (9 months) as second-line therapy in patients who had not received prior thalidomide or bortezomib treatment. In a phase-2 study, Palumbo *et al.*^26^ found a somewhat longer PFS (17 months) in 62 patients receiving thalidomide–dexamethasone as second-line treatment, and most of the patients (97%) had initially received ASCT. Stadtmauer *et al.*^16^ analyzed two large phase-3 studies of lenalinomide–dexamethasone in relapsed or refractory myeloma (MM-009 and MM-010) with respect to second-line treatment and found PFS of 14.1 months and OS of 42.0 months, and here 67% of these patients had received initial ASCT. Thus, our results are better than the findings in most published studies on second-line treatment in relapsed myeloma patients. However, one must be cautious when comparing different studies, as the selection of patients has a great impact on the prognosis, as indicated by the significance of the first PFS after initial treatment as documented in the present study.

In this study, we did not find any significant difference in PFS or TNT after the salvage ASCT compared with the first ASCT, which was better than expected. This comparison may be problematic as death is included as an event, but obviously it could only be an issue after salvage ASCT. However, this would introduce a bias for better median PSF and TNT than average after an initial ASCT. On the other hand, the 49 patients who completed salvage ASCT were selected, but still they were their own controls. Therefore, the results are interesting and important.

In a recent retrospective analysis of salvage second ASCT, Michaelis *et al.*^1^ reported registry data from 187 patients reported to the Center for International Blood and Bone Marrow Transplant Research (CIBMTR).

The authors also reviewed five other retrospective studies on salvage ASCT in multiple myeloma. The results showed a medium PFS of 8.5–16.4 months and OS of 19–53 months where the time to relapse after the initial ASCT had a major impact on the PFS after the salvage ASCT. The conclusion was that salvage ASCT should be considered only in patients who relapse/progress later than 1.5 years after the initial ASCT. This is in accordance with the review by Atanackovic and Schilling.^8^ Our results show a little longer PFS and OS after salvage ASCT with bortezomib in bortezomib-naïve patients and a median OS of about 10 months for patients with PFS <1.5 years after the initial ASCT, and this is longer than 6 months OS reported by Alvares *et al.*^6^ The improved depth of response induced by reinduction with bortezomib–dexamethasone may partly be responsible for the effect by salvage ASCT in the present study of bortezomib-naïve patients. However, as the treatment of younger newly diagnosed myeloma patients still improves with inclusion of new drugs in the induction therapy and by inclusion of consolidation and/or maintenance therapy, the future use of salvage ASCT should still be subject for prospective randomized clinical studies where the treatment is adjusted to the former treatment of the patients.

In the only prospective randomized study published most recently, Cook *et al.*^9^ compared salvage HDM with ASCT to weekly oral cyclophosphamide in patients who had not progressed during reinduction with PAD (bortezomib–doxorubicin–dexamethasone). They showed improved PFS of 19 months compared with 11 months in the cyclophosphamide group and comparable to the findings in the present study of 19.3 months.

In our study, the quality of the response shows a trend to improve by increasing the percentage of VGPR or CR/nCR from 54 to 66.5, and in one-third of the patients VGPR was achieved before HDM–bortezomib. In the retrospective study of Center for International Blood and Bone Marrow Transplant Research (CIBMTR), the chemosensitivity and disease status had no effect on outcomes after the salvage ASCT.^1^ In addition, the response rates were comparable to the phase-3 study showing 59% VGPR or better.^9^

The combination of bortezomib and HDM was generally well tolerated. The expected side effects were mainly of low grade. Some patients experienced a flare-up of the neurological side effects following salvage HDM–bortezomib with stem cell support, and in one patient the symptoms seem to have become chronic. In all other cases the neurological symptoms had disappeared after 6−12 months. In the phase-1/2 trial of the combination of bortezomib and ASCT, Lonial *et al.*^21^ did not report any neurological adverse event, possibly because only 60% of their patients had received bortezomib before and they only used a single dose in connection with ASCT. The neurotoxicity might be further reduced by administering bortezomib subcutaneously;^27^ however, this was not approved at the time of the present study. Four of the 53 patients never went on with ASCT, one because of early death, one because of toxicity and two because of progressive disease. The retrospective studies gave no information of how many patients were excluded before planned salvage ASCT.

In conclusion, the findings in our study support the use of salvage HDM–bortezomib after reinduction with bortezomib and dexamethasone induction at least in bortezomib-naive patients. Our study shows an efficacy comparable to the recent prospective randomized study that documented the efficacy of salvage HDM compared with cyclophosphamide after induction with the bortezomib-containing regimen PAD. More prospective randomized studies are needed to find the optimal place and regimen for salvage HDM eventually stratified for the initial induction therapies.

## Figures and Tables

**Table 1:** Baseline characteristic of 53 patients with multiple myeloma planned for salvage high-dose melphalan with ASCT at first symptomatic relapse after initial ASCT

*Baseline characteristics*		
	*Median*	*Range*
Age (years)	60	36–70
Gender	Female 21 (40%)	
	Male 32 (60%)	
M-protein	IgG 36 (73%)	
	IgA 9 (18%)	
	IgD 1 (2%)	
	Light chain only three (6%)	
	Unknown 4	
		
*Initial induction*		
VAD	10	
CTX/Dex	43	
Maintenance		
IFN^a^	7	
None	46	
PFS (I) (months)	25.3	3.5–112.3
TNT (I) (months)	29.1	4.8–112.3
ISS	I 19 (36%)	
	II 12 (23%)	
	III 10 (19%)	
	NA 12 (23%)	

Abbreviations: ASCT=autologous stem cell support; CTX/Dex=cyclophosphamide and dexamethasone (two series followed by CTX-G-CSF for mobilizing peripheral stem cells); ISS=international staging system; PFS (I)=progression-free survival after initial ASCT; TNT (I)=time to next treatment after initial ASCT; VAD=vincristin, adriamycin, dexamethasone (three series followed by CTX-G-CSF for mobilizing peripheral stem cells).

a Interferon 2α maintenance three times a week.

**Table 2:** Response rates^a^

	*CR/nCR*	*VGPR*	*PR*	*SD*	*PD*	*NE*
Induction (I)	2 (4%)	0 (0%)	30 (61%)	15 (31%)	1 (2%)	1 (2%)
ASCT (I)	12 (25%)	14 (28%)	23 (47%)	0%	0	0%
After reinduction	10 (20%)	6 (12%)	17 (35%)	13 (27%)	1 (2%)	2 (4%)
ASCT (II)	17 (35%)	15 (30,5%)	15 (30,5%)	1 (2%)	0	1 (2%)

a Response rates after salvage high-dose melphalan with bortezomib and stem cell support compared with the initial response rates and after bortezomib–dexamethsone reinduction with three courses of bortezomib–dexamethasone at first relapse compared with the response to initial induction with VAD or CTX-Dex.

**Table 3:** Neurotoxicity after induction therapy with bortezomib–dexamthasone (*N*=51—two patients could not be evaluated) and after high-dose melphalan with bortezomib and stem cell support (*N*=49)

	*Grade 0*	*Grade 1*	*Grade 2*	*Grade 3*	*Grade 4*
*After induction (Btz-Dex)*					
Neuropatic pain	43	7	0	1	0
Sensory neuropathy	26	21	3	1	0
					
*After HDM-Btz*					
Neuropatic pain	45	0	2	2	0
Sensory neuropathy	40	4	2	3	0

Abbreviations: Btz-Dex=bortezomib–dexamethasone; HDM-Btz=bortezomib–high-dose melphalan.

Neurotoxicity maximal grade (according to National Cancer Institute Common Toxicity Criteria for Adverse Events (NCI CTCAE), version 3.0 (http://ctep.cancer.gov/forms/CTCAEv3.pdf)).

**Table 4:** Adverse event (maximal grade) after high-dose melphalan–bortezomib with stem cell support according to National Cancer Institute Common Toxicity Criteria for Adverse Events (NCI CTCAE)

*Adverse event*	*Grade 0*	*Grade 1*	*Grade 2*	*Grade 3*	*Grade 4*
Diarrhea	24 (49%)	14 (29%)	5 (10%)	6 (12%)	0
Nausea	21 (43%)	11 (22%)	14 (29%)	3 (6%)	0
Mucositis	35 (72%)	6 (12%)	5 (10%)	3 (6%)	0
Anorexia	44 (90%)	2 (4%)	1 (2%)	2 (4%)	0
Fatigue	44 (90%)	3 (6%)	1 (2%)	1 (2%)	0
Constipation	45 (92%)	2 (4%)	2 (4%)	0	0
Vomiting	40 (82%)	7 (14%)	2 (4%)	0	0
Pyrexia	30 (61%)	4 (8%)	6 (12%)	8 (17%)	1 (2%)
Infection	34 (70%)	1 (2%)	5 (10%)	7 (14%)	2 (4%)
Fungal infection	46 (94%)	1 (2%)	2 (4%)	0	0
Dizziness	44 (90%)	5 (10%)	0	0	0
Hypotension	45 (92%)	1 (2%)	3 (6%)	0	0
Hypoxia	47 (96%)	1 (2%)	1 (2%)	0	0
Pain	40 (82%)	5 (10%)	3 (6%)	1 (2%)	0
Neuropathic pain	44 (90%)	2 (4%)	1 (2%)	2 (4%)	0
Sensory neuropathy	41 (84%)	5 (10%)	2 (4%)	1 (2%)	0
Muscle cramps	48 (98%)	1 (2%)	0	0	0
Cerebral hemorrhage	48 (98%)	0	0	0	1 (2%)
Insomnia	48 (98%)	1 (2%)	0	0	0
Low potassium	44 (90%)	4 (8%)	1 (2%)	0	0
Atrial fibrillation	47 (96%)	0	1 (2%)	0	1 (2%)
Renal	48 (98%)	1 (2%)	0	0	1
Psychiatric	42 (86%)	3 (6%)	2 (4%)	2 (4%)	0
Rash	42 (86%)	6 (12%)	1 (2%)	0	0

Version 3.0 (http://ctep.cancer.gov/forms/CTCAEv3.pdf).

## Notes

Janssen-Cilag supplied the study with free bortezomib and supported the expenses for conducting the study with a grant. Janssen-Cilag, together with Celgen and Mundipharma, are sponsors of The Nordic Myeloma Study Group. During the trial, the Nordic Myeloma Study Group has also received funding from the Nordic Cancer Union. P. Gimsing has received lecture honaria from Amgen. N. Abildgaard has received research grants Celgene and Janssen-Cilag. UH Mellqvist has received lecture honoraria from Celgene, Jansen, Mundipharma and Amgen. All remaining authors have declared no conflicts of interest.
